# Early Vitamin C, Hydrocortisone, and Thiamine Treatment for Septic Cardiomyopathy: A Propensity Score Analysis

**DOI:** 10.3390/jpm11070610

**Published:** 2021-06-28

**Authors:** Min-Taek Lee, Sun-Young Jung, Moon Seong Baek, Jungho Shin, Won-Young Kim

**Affiliations:** 1 Department of Global Innovative Drugs, The Graduate School of Chung-Ang University, Chung-Ang University, Seoul 06974, Korea; come_410@naver.com (M.-T.L.); jsyoung@cau.ac.kr (S.-Y.J.); 2 College of Pharmacy, Chung-Ang University, Seoul 06974, Korea; 3 Division of Critical Care Medicine, Department of Internal Medicine, Chung-Ang University Hospital, Chung-Ang University College of Medicine, Seoul 06973, Korea; wido21@cau.ac.kr; 4 Division of Nephrology, Department of Internal Medicine, Chung-Ang University Hospital, Chung-Ang University College of Medicine, Seoul 06973, Korea; junghoshin@cau.ac.kr

**Keywords:** ascorbic acid, cardiomyopathies, hydrocortisone, sepsis, septic shock, thiamine

## Abstract

This study investigated the effectiveness of early vitamin C, hydrocortisone, and thiamine among patients with septic cardiomyopathy. In total, 91 patients with septic cardiomyopathy received a vitamin C protocol in September 2018–February 2020. These patients were compared to 75 patients with septic cardiomyopathy who did not receive a vitamin C protocol in September 2016–February 2018. Relative to the control patients, the treated patients were older and more likely to require mechanical ventilation. The vitamin C protocol was associated with a lower risk of intensive care unit mortality in the propensity score (PS)-matched cohort (aHR: 0.55, 95% CI: 0.30–0.99) and inverse probability of treatment weighting-matched cohort (aHR: 0.67, 95% CI: 0.45–1.00). In the PS-matched cohort (59 patients per group), the vitamin C protocol was associated with decreased values for vasopressor dosage, C-reactive protein concentration, and the Sequential Organ Failure Assessment score during the 4-day treatment period. Patients who started the vitamin C protocol within 2 h after diagnosis (vs. 2–8 h or ≥8 h) had the highest vasopressor weaning rate and the lowest mortality rate. These results suggest that early treatment using a vitamin C protocol might improve organ dysfunction and reduce mortality among patients with septic cardiomyopathy.

## 1. Introduction

Sepsis is a dysregulated host response to infection that involves various organs [1]. Septic cardiomyopathy is one manifestation of septic shock and is associated with a mortality rate of up to 50% [2]. The pathogenesis of septic cardiomyopathy involves a complex interaction between chemical mediators and mitochondrial dysfunction [3,4,5], although no specific treatment exists. Morelli et al. reported that β-blockers might improve survival outcomes for patients with septic shock [6], although the control group in that study had a high mortality rate. Another potential treatment is levosimendan, which is a calcium sensitizer that has inotropic and vasodilating properties [7], although it was not associated with improved organ dysfunction or a lower mortality rate among patients with sepsis or cardiac dysfunction [8].

Vitamin C exerts anti-inflammatory effects, maintains endothelial function, and facilitates the production of catecholamines and vasopressin [9,10]. Thus, vitamin C might be useful for treating septic cardiomyopathy. Furthermore, vitamin C and corticosteroids could have additive effects [11], and thiamine and vitamin C may act synergistically to limit oxidative injury [12]. Several randomized trials have evaluated the effects of vitamin C, hydrocortisone, and thiamine treatment (the vitamin C protocol) for sepsis [13,14,15,16], although it was not associated with significantly improved outcomes. However, these studies were limited by delayed vitamin C administration and heterogeneous patient populations, and there is some evidence that different septic phenotypes may respond differently to the vitamin C protocol [17]. Therefore, the present study evaluated whether the vitamin C protocol, which was routinely used at our center starting in September 2018, could improve organ function and mortality among patients with septic cardiomyopathy. We used a before-and-after design to compare patients who were admitted in the intensive care unit (ICU) between September 2016 and February 2018 (the “before” cohort) and between September 2018 and February 2020 (the “after” cohort). The adjustment for the covariate balancing was performed using propensity score (PS) analysis.

## 2. Materials and Methods

### 2.1. Study Design and Patients

This study was a retrospective analysis of patients with septic cardiomyopathy who were admitted to the medical ICU of a tertiary care hospital in Seoul, Korea. The treatment group was defined as patients who were admitted between September 2018 and February 2020. The control group was defined as those who were treated in the same ICU between September 2016 and February 2018 and did not receive the vitamin C protocol. The exclusion criteria were age < 19 years, no diagnosis of septic shock, and/or those who could not be resuscitated or intubated. We did not exclude patients with a history of chronic heart failure if the new-onset failure was characterized by echocardiography and did not suggest coronary artery disease, valvular heart disease, or other cardiomyopathies. The primary outcome of the study was ICU mortality. Secondary outcomes included vasopressor weaning, vasopressor-free days at day 28, ventilator weaning, ventilator-free days at day 28, and superinfection. The retrospective study protocol was approved by the Institutional Review Board of Chung-Ang University Hospital (1820-006-353). Written informed consent was waived because it was an observational study.

### 2.2. Septic Cardiomyopathy

Echocardiographic identification of septic cardiomyopathy included decreased ventricular contractility, left ventricular (LV) dilation under normal or low filling pressure, and right ventricular dysfunction and/or LV dysfunction with a reduced response to fluid infusion [18]. However, screening for septic cardiomyopathy was not routinely performed at our center. Alternatively, cardiac troponin I (cTnI) and brain natriuretic peptide (BNP) were collected among patients without echocardiographic data. Elevations of these markers are associated with cardiac dysfunction in sepsis [19,20]. Septic cardiomyopathy was identified based on concentrations above the upper limit of normal for cTnI (≥34 ng/L) and BNP (≥100 pg/mL), as previously described [8].

### 2.3. Treatment Protocol

The vitamin C protocol was initiated in all septic patients with persistent hypotension despite adequate fluid resuscitation, which required vasopressors. Patients received intravenous vitamin C 1.5 g every 6 h for 4 days, hydrocortisone 50 mg every 6 h for 7 days, and thiamine 200 mg every 12 h for 4 days [17]. Only corticosteroids were administered in the control group (September 2016–February 2018) at the discretion of attending ICU clinicians. All patients were managed in accordance with the recommendations from the Surviving Sepsis Campaign guidelines [21]. Apart from the use or non-use of the vitamin C protocol, there were no significant changes in our ICU protocols and patient population between the two study periods.

### 2.4. Data Collection and Definitions

The following baseline variables were collected from the Chung-Ang University Hospital Information System: age, sex, body mass index, comorbidities, cause of sepsis, nosocomial infection, acute respiratory distress syndrome (ARDS), Sequential Organ Failure Assessment (SOFA) score [22], mechanical ventilation, and renal replacement therapy. The times of septic shock onset and starting the vitamin C protocol were extracted. In addition, data were extracted for the first 4 days after ICU admission regarding vital signs, laboratory data, vasopressor dosage, SOFA score, fluid intake, and output volumes. The daily vasopressor dosage was obtained at 07:00 and converted to the norepinephrine equivalent dose, which was calculated as (norepinephrine [μg/min]) + (dopamine [μg/kg/min] ÷ 2) + (epinephrine [μg/min]) + (phenylephrine [μg/min] ÷ 10) [23]. To minimize survivorship bias, a maximum SOFA score of 24 was assigned to patients with missing values because of death, and the last observation carried forward (LOCF) approach was used for patients who were discharged [24]. The LOCF approach was also used for missing entries regarding C-reactive protein (CRP) concentration, lactate concentration, and norepinephrine equivalent dose. Experienced cardiologists reviewed the echocardiographic parameters (LV ejection fraction, mitral *E* wave, mitral *E′* wave, *E/E′* ratio, and velocity time integral). Atrial or ventricular arrhythmias requiring chemical or electrical cardioversion were also collected. Patients fulfilling the Sepsis-3 consensus definition of septic shock [1] were recruited, although some patients with suspected infection who had serum lactate concentrations of <2 mmol/L were included because they had persistent hypotension that required high-dose vasopressors. Immunosuppression was identified if the patient was diagnosed with human immunodeficiency virus infection, malignancy, or severe neutropenia, or was receiving immunosuppressive therapy. The consensus definition was used to identify ARDS [25]. Vasopressor weaning was defined when the patient maintained normal blood pressure for ≥48 h without any vasopressor support. Ventilator weaning was identified when the patient was free from any form of ventilator support for ≥48 h. Superinfection was defined if a new microbiological infection occurred at ≥48 h after admission.

### 2.5. Statistical Analysis

Continuous variables were presented as median (interquartile range, IQR) or as mean ± standard deviation and were compared using the Mann–Whitney *U* test or Student’s *t*-test. The Kruskal–Wallis test was used to compare continuous variables among three groups. Categorical variables were presented as a number (percentage) and were compared using the chi-squared test or Fischer’s exact test.

We used PS analysis to account for baseline differences in the probabilities of receiving the vitamin C protocol [26]. The factors that were used to calculate the PSs are described in Table S1. The model’s discrimination was evaluated using the *c*-statistic (*c* = 0.75), and its calibration was evaluated using the Hosmer-Lemeshow test (chi-square = 10.75; *p* = 0.22). A Cox regression model was used to evaluate the association between the vitamin C protocol and ICU mortality. In the unmatched cohort, the individual PSs and lactate concentration (the variable associated with ICU mortality that was not used to calculate the PS) were incorporated into the model as the covariates to calculate the PS-adjusted hazard ratio (HR). Patients in the treatment and control groups were matched 1:1 using nearest neighbor matching based on a greedy matching algorithm [27]. Standardized mean differences were calculated before and after the matching to confirm whether the covariates were balanced. After all PS matches were performed, the inter-group differences were evaluated using the paired *t*-test or the Wilcoxon signed rank test for continuous variables and using the chi-squared test or Fisher’s exact test for categorical variables. Inverse probability of treatment weighting (IPTW) analysis was also performed using the PS-based weights, with trimming of the non-overlap regions to ensure that patients had a non-zero probability of receiving either treatment [28]. Cox regression analysis with ICU mortality as the outcome was performed for the PS-matched and IPTW-matched treatment and control groups.

The time-response relationship between vitamin C protocol administration and clinical outcomes was evaluated using three intervals between shock onset and the start of treatment (<2 h, 2–8 h, and ≥8 h). The cut-off values (2 h and 8 h) defined the upper and lower quartiles of the treatment group. All tests were two-tailed, and differences were considered statistically significant at *p*-values of <0.05. All analyses were performed using IBM SPSS software (version 26.0; IBM Corp., Armonk, NY, USA) and SAS software (version 9.4; SAS Institute, Cary, NC, USA).

## 3. Results

During the treatment period, 196 eligible patients received the vitamin C protocol, and 91 of these patients were diagnosed with septic cardiomyopathy (echocardiography: 44 patients, cardiac biomarkers: 47 patients). These 91 patients were defined as the treatment group. During the control period, 228 eligible patients were admitted to the ICU, and 75 of these patients were diagnosed with septic cardiomyopathy (echocardiography: 34 patients, cardiac biomarkers: 41 patients). These 75 patients were defined as the control group. Baseline characteristics and clinical outcomes of the unmatched treatment and control groups are shown in Tables S2 and S3, respectively. The treated patients were more likely to be older, male, and have a lower body mass index. The treated patients also tended to be more likely to require mechanical ventilation.

### 3.1. Propensity Score Analysis

The PS matching created 59 pairs of patients who did or did not receive the vitamin C protocol. Table 1 shows the baseline characteristics of the matched treatment and control groups. The imbalances between the treatment and control groups were considerably reduced after the matching (Figure S1). The primary cause of sepsis was pneumonia in both groups. There were no significant differences between the groups in terms of the SOFA score, vasopressor dosage, LV systolic function, and arrhythmia (Table 2 shows the detailed echocardiographic parameters). Similar echocardiographic findings were observed between the groups when we evaluated patients with chronic heart failure, although patients with chronic lung disease had significantly lower cardiac index in the treatment group (Table S4). Among patients without echocardiographic data, there were no significant between-group differences in the median cTnI concentrations (113 ng/L (IQR: 71–259 ng/L) vs. 189 ng/L (IQR: 87–527 ng/L); *p* = 0.20) and the median BNP concentrations (387 pg/mL (IQR: 268–876 pg/mL) vs. 280 pg/mL (IQR: 144–678 pg/mL); *p* = 0.17). The median duration of vitamin C treatment was 2 days (IQR: 2–3 days), and most of the patients started treatment at 2–11 h (median: 4 h) after shock onset.

In the matched cohort, the ICU mortality rates were 32% (19/59 patients) in the treatment group and 46% (27/59 patients) in the control group (*p* = 0.13) (Table 3). Furthermore, in the matched cohort, treated patients had significantly more ventilator-free days at day 28 and tended to have less superinfection rate. There were no significant differences in terms of ICU mortality, vasopressor-free days at day 28, and superinfection rate when the patients were stratified according to the presence of chronic heart failure (Table S5). Among the 57 patients who received echocardiography, 24 patients (42%) received follow-up evaluation, and the recovery group tended to have lower ICU mortality rate and more vasopressor-free days at day 28 (Table S6). However, the proportion of recovered patients (70% vs. 71%; *p* > 0.99) and follow-up LV systolic function (ejection fraction: 50% (IQR: 29–55%) vs. 53% (IQR: 46–59%); *p* = 0.46) were not significantly different between the treatment and control groups. The control group included 13 patients (22%) who received corticosteroids, with a median dose (hydrocortisone equivalents) of 150 mg/day (IQR: 30–300 mg/day) and a median duration of 3 days (IQR: 1–9 days). A comparison of the treatment group, the corticosteroid-treated control group, and the control group, which did not receive corticosteroids, failed to detect significant differences in terms of ICU mortality, vasopressor-free days at day 28 and superinfection rate (Table S7). However, the treatment group had significantly more ventilator-free days at day 28.

Use of the vitamin C protocol was associated with a significantly lower risk of mortality in the unadjusted analysis (unadjusted HR: 0.57, 95% confidence interval (CI): 0.35–0.93; *p* = 0.02) (Table 4). The Cox regression model, which was adjusted for the PS plus the lactate concentration, revealed that the vitamin C protocol tended to be associated with a lower risk of mortality in the unmatched cohort (adjusted HR: 0.60, 95% CI: 0.34–1.08; *p* = 0.09). Moreover, the vitamin C protocol was associated with significantly lower risks of mortality in the PS-matched cohort (adjusted HR: 0.55, 95% CI: 0.30–0.99; *p* = 0.047) and in the IPTW-matched cohort (adjusted HR: 0.67, 95% CI: 0.45–1.00; *p* = 0.048).

### 3.2. Changes in Clinical Parameters

**Figure 1:**
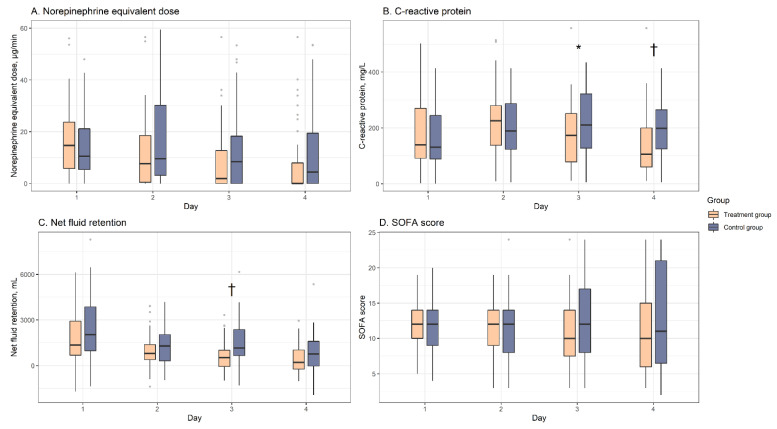
**A:** Vasopressor dosages (norepinephrine equivalents), **B:** CRP concentrations, **C:** net fluid retentions, **D:** SOFA scores during the 4-day study period. The center lines indicate the median values, the box tops and bottoms indicate the interquartile ranges, error bars indicate the overall ranges, and the dots indicate outliers. CRP: C-reactive protein; SOFA: Sequential Organ Failure Assessment. * *p* < 0.05, † *p* < 0.01 when the treatment and control groups were compared by the Mann–Whitney *U* test or Student’s *t*-test.

In the matched cohort, the 4-day changes in the clinical parameters are shown in Figure 1 and Table S8. The vasopressor dosage (norepinephrine equivalents) decreased over time for the treatment and control groups, although the decrease at day 4 was significantly greater in the treatment group (median: −8.4 μg/min (IQR: −21.0 to 0 μg/min) vs. −4.1 μg/min (IQR: −10.8 to 2.0 μg/min); *p* = 0.04). The median CRP concentration only decreased significantly in the treatment group (−25 mg/L (IQR: −116 to 46 mg/L) vs. 0 mg/L (IQR: −30 to 122 mg/L); *p* = 0.004). During the 4-day period, the treatment group generally exhibited less fluid retention. There was no significant difference in the groups’ SOFA scores at day 1, although the decrease at day 4 tended to be greater in the treatment group (median: −2 (IQR: −5 to 1) vs. −1 (IQR: −3 to 4); *p* = 0.09).

### 3.3. Vasopressor Weaning and ICU Mortality

**Figure 2:**
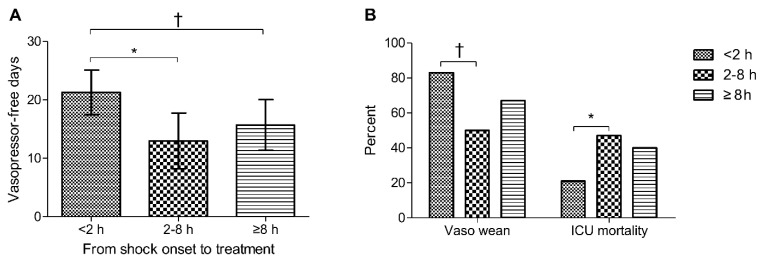
(**A**) Vasopressor-free days at day 28, (**B**) vasopressor weaning, and ICU mortality according to timing of the vitamin C protocol. Error bars indicate the 95% confidence intervals. ICU: intensive care unit. * *p* < 0.05, † *p* < 0.01 when the groups were compared by the Mann–Whitney *U* test or Student’s *t*-test for continuous variables and by the chi-squared test or Fisher’s exact test for categorical variables.

Of the 91 patients in the unmatched cohort, 29 started the vitamin C protocol at <2 h after shock onset, 32 at 2–8 h, and 30 at ≥8 h. Patients who started the protocol at <2 h after shock onset (vs. 2–8 h or ≥8 h) had a higher mean number of vasopressor-free days at day 28 (21.3 ± 10.0 days vs. 13.0 ± 13.2 days vs. 15.7 ± 11.6 days) and a higher vasopressor weaning rate (83% vs. 50% vs. 67%) (Figure 2). Early administration of the vitamin C protocol was associated with a decreased rate of ICU mortality (21% vs. 47% vs. 40%).

## 4. Discussion

This study revealed that, after adjusting for baseline imbalances in the treatment and control groups’ characteristics, the vitamin C protocol was associated with a significantly lower risk of mortality. This result was also supported by the treatment group, having significant decreases in vasopressor dosage and CRP concentration (an inflammation marker). These effects might be driven by early (<2 h) initiation of treatment.

Preclinical studies have shown that increased oxidative stress in cardiomyocytes and subsequent injury may induce septic cardiomyopathy [29]. Hao et al. showed that vitamin C decreased myocardial oxidant injury, attenuated apoptosis, and preserved the functional integrity of mitochondria [30]. In addition, both vitamin C and hydrocortisone protect the vascular endothelium and reduce the ischemia-reperfusion injury [31,32]. Thiamine, vitamin C, and corticosteroids may act synergistically to reverse the organ dysfunction in sepsis [33]. These findings imply that the vitamin C protocol may have a protective role in septic cardiomyopathy. In the present study, the treatment group had significant decreases in vasopressor dosage. However, there is no biologically plausible explanation for this finding, as we did not have any data on the evolution of cardiac or vascular dysfunction.

An explanation is needed for why the vitamin C protocol was not associated with the overall ICU mortality rate in the unmatched analysis but was significantly associated with the mortality rate in the Cox regression model. First, the treatment group was older and more likely to require mechanical ventilation, relative to the control group, and these covariates were considered important in our Cox regression model, PS generation model, and IPTW-matched analysis. Thus, it seems reasonable that the relationship between the vitamin C protocol and ICU mortality became apparent after adjusting for these factors. Second, the early mortality rates were high in the treatment and control groups, with 4-day mortality rates of 20% (18/91 patients) in the treatment group and 31% (23/75 patients) in the control group (*p* = 0.11). Furthermore, in the matched cohort, the 4-day mortality rates were 22% (13/59 patients) in the treatment group and 31% (18/59 patients) in the control group (*p* = 0.30). It is possible that the Cox model for time to death reflected a short-term effect of the vitamin C protocol on mortality, given that the protocol only involved 4 days with vitamin C administration. Thus, the benefits of vitamin C treatment might be clearer if the outcome measurement was confined to the days on which it was administered, as re-analysis of the CITRIS-ALI trial data revealed that vitamin C treatment was associated with an 81% lower mortality rate during the 4-day treatment period [34].

Survivorship bias should also be considered to accurately interpret the results of clinical trials that involve critically ill patients [35]. For example, the CITRIS-ALI trial revealed no significant difference in the SOFA score change at 96 h between the vitamin C and placebo groups [36]. However, 64 of 167 patients (38%) were excluded based on missing values because of death and early discharge. Thus, the survivors with relatively severe organ dysfunction in the vitamin C group might have been compared to survivors with a relatively better condition in the placebo group. In addition, secondary analysis that assigned a SOFA score of 20 to patients who died and a score of 0 to patients who were discharged revealed a significant difference in the SOFA scores at 96 h (*p* = 0.03) [37]. In the present study, we assigned a SOFA score of 24 to deceased patients and used the LOCF approach for discharged patients. The results revealed a near-significant difference between the treatment and control groups in the SOFA score change at day 4. Furthermore, this finding is consistent with the treatment group having significant decreases in vasopressor dosage and CRP concentration, which supports the hypothesis that the vitamin C protocol might help improve organ dysfunction and inflammation among patients with septic cardiomyopathy.

Previous randomized trials of vitamin C treatment for sepsis may also be limited by delayed treatment initiation. For example, the CITRIS-ALI trial required fully developed ARDS, and most patients were in the advanced stage of sepsis at enrollment [36]. Furthermore, the median times from fulfilling the eligibility criteria to vitamin C administration were 12.1 h in the VITAMINS trial, 14.5 h in the ACTS trial, and 14.7 h in the VICTAS trial [13,15,16]. However, retrospective studies have indicated that starting treatment at <6 h after sepsis presentation can influence patient outcomes [38,39]. The ORANGES trial also identified quicker shock reversal in the vitamin C protocol group, which was independent of the effects of corticosteroids in the placebo group [14], and most patients received their first treatment within 3–14 h after presenting to the emergency department. In the present study, the vitamin C protocol was initiated at a median interval of 4 h after shock onset, and large treatment effects were associated with early therapy initiation (<2 h). Therefore, early administration of vitamin C, antibiotics, and vasopressors may help clarify the treatment-specific effects and minimize variations in the standard care for septic shock. Nevertheless, further studies are needed to validate the temporal benefits of the vitamin C protocol.

The survival outcomes of previous trials might also be influenced by differences between specific subgroups. It has been suggested that the lack of a clear benefit in the trial of levosimendan for septic shock was related to the fact that not all patients had cardiac dysfunction [40]. Moreover, vitamin C may be most beneficial for patients with the most severe disease [41]. In this context, all of our patients had severe cardiac dysfunction (all were receiving vasopressors, and the median SOFA scores were 11–12) and we observed that the treated patients were likely to have more ventilator-free days at day 28. This finding is consistent with a recent meta-analysis that indicated that vitamin C might decrease the duration of mechanical ventilation among critically ill patients [41]. This relationship is interesting and should be explored, given that high-dose vitamin C may help reduce mortality rates in cases of severe pneumonia, including cases that involve coronavirus disease 2019 [42,43]. Patients with sepsis are at high risk of atrial and ventricular arrhythmias [44]. Moreover, a recent study showed a significant association between sepsis-induced LV dysfunction and arrhythmias and circulating histones, which can be released into the bloodstream due to excessive inflammation and cellular death [45]. Our study revealed up to 27% (32/118 patients) in the unmatched cohort who experienced arrhythmias requiring cardioversion. Therefore, additional studies are needed to identify patient groups that may benefit from this treatment strategy.

This study has several limitations. First, the single-center retrospective analysis of temporally separated treatment and control groups is prone to bias, which cannot be completely excluded, although we used PS matching, IPTW, and Cox regression analyses to reduce the contribution of treatment selection bias. Second, the small number of patients might have limited the power of the analyses to detect significant effects that could be attributed to the vitamin C protocol. Third, echocardiographic parameters were only available for 48% of the patients, and cardiac biomarkers were used to identify septic cardiomyopathy in the remaining patients. In addition, the number of echocardiography was further reduced to 20% for follow-up examination. The conclusions drawn in this study were not based on a unified method for screening of septic cardiomyopathy and may be subject to bias. Moreover, troponin elevation may be affected by multiple organs and pathophysiologic pathways, while BNP elevation may be confounded by respiratory failure, renal dysfunction, and atrial fibrillation [46]. Fourth, patients who received corticosteroids were not excluded from the control group to evaluate whether the combination therapy was more effective than corticosteroid monotherapy. Corticosteroid treatment was administered at the discretion of attending ICU clinicians to 22% of the control group (13/59 patients), and there is a possibility that the steroid treatment affected the outcomes in the control group. However, we failed to detect significant differences in terms of ICU mortality, vasopressor-free days at day 28, and superinfection rate when we compared control patients who did or did not receive corticosteroids. Fifth, the median duration of vitamin C treatment (2 days) was shorter than the recommended 4-day protocol by early death or discharge, and the vitamin C concentrations were not measured. Thus, it is possible that the vitamin C concentrations were suboptimal, which might have reduced the 4-day changes in the treatment group’s clinical parameters.

## 5. Conclusions

In conclusion, combination treatment using vitamin C, hydrocortisone, and thiamine for patients with septic cardiomyopathy is associated with a lower risk of mortality. It also reduced values for vasopressor dosage, CRP concentration, and SOFA score during the first 96 h after shock onset. Furthermore, earlier initiation of this treatment seems to be linked to a greater therapeutic effect. However, validation in a large prospective study is needed to confirm our results.

## Figures and Tables

**Table 1:** Baseline characteristics of the groups after matching.

	Treatment Group (*n* = 59)	Control Group (*n* = 59)	*p*
Age, years	77 (71–83)	76 (66–82)	0.51
Male sex	32 (54)	26 (44)	0.27
Body mass index, kg/m^2^	21.3 (19.5–23.4)	21.0 (18.5–23.5)	0.63
Comorbidities			
Diabetes	21 (36)	26 (44)	0.35
Chronic heart failure	6 (10)	6 (10)	>0.99
Chronic lung disease	9 (15)	9 (15)	>0.99
Liver cirrhosis	6 (10)	6 (10)	>0.99
Chronic kidney disease	11 (19)	13 (22)	0.65
Malignancy	12 (20)	8 (14)	0.33
Immunosuppression	12 (20)	12 (20)	>0.99
Cause of sepsis			
Pneumonia	26 (44)	29 (49)	0.58
Urosepsis	14 (24)	17 (29)	0.53
Gastrointestinal/biliary	14 (24)	17 (29)	0.53
Nosocomial infection	20 (34)	18 (31)	0.69
ARDS	5 (8)	6 (10)	0.75
SOFA score	12 (10–14)	12 (9–14)	0.93
Mechanical ventilation	40 (68)	35 (59)	0.34
Renal replacement therapy	24 (41)	23 (39)	0.85
Vital signs & laboratory data			
Body temperature, °C	37.2 (36.8–38.1)	37.3 (36.8–37.9)	0.72
Mean arterial pressure, mmHg	61 (54–66)	60 (53–67)	0.68
Respiratory rate, breaths/min	28 (25–33)	28 (24–34)	0.60
Bicarbonate, mEq/L	17.5 (13.8–19.2)	16.9 (13.1–20.8)	0.49
Creatinine, mg/dL	1.6 (1.1–2.2)	1.7 (1.1–2.3)	0.78
Platelet count, 1000/mm^3^	132 (79–197)	117 (61–178)	0.48
Total bilirubin, mg/dL	0.8 (0.5–1.8)	0.7 (0.4–1.2)	0.17
C-reactive protein, mg/L	140 (91–274)	132 (89–248)	0.84
Lactate, mmol/L	4.7 (2.5–6.7)	3.5 (1.9–6.9)	0.33
Cardiac troponin I, ng/L	163 (62–722)	211 (91–737)	0.29
Brain natriuretic peptide, pg/mL	458 (242–943)	318 (144–988)	0.48
Norepinephrine equivalent dose, μg/min	14.7 (5.4–24.7)	10.5 (5.4–21.2)	0.67
Echocardiography (*n* = 29/*n* = 28) ^1^			
Ejection fraction, %	34 (31–42)	39 (32–45)	0.67
Arrhythmia	20 (34)	12 (20)	0.10

Data are presented as number (%) or median (interquartile range). The *p*-values were calculated using the paired *t*-test or the Wilcoxon signed rank test for continuous variables and using the chi-squared test or Fisher’s exact test for categorical variables. ARDS: acute respiratory distress syndrome; SOFA: Sequential Organ Failure Assessment. ^1^ Echocardiography was performed for 29 patients in the treatment group and for 28 patients in the control group.

**Table 2:** Echocardiographic parameters.

	Treatment Group (*n* = 29)	Control Group (*n* = 28)	*p*
LV ejection fraction, %	34 (31–42)	39 (28–45)	0.79
Mitral *E* wave, cm/s	80.0 (62.0–95.0)	65.5 (51.5–89.0)	0.15
Mitral *E’* wave, cm/s	5.0 (4.0–7.0)	5.0 (4.0–6.2)	0.54
*E/E′* ratio	16.0 (11.2–17.8)	12.1 (10.4–17.0)	0.39
Stroke volume, mL	24 (22–35)	27 (24–33)	0.59
Cardiac output, L/min	2.8 (2.3–3.1)	3.0 (2.3–3.7)	0.24
Cardiac index, L/min/m^2^	1.7 (1.5–1.9)	2.0 (1.5–2.4)	0.08

Data are presented as the median (interquartile range). The *p*-values were calculated using the Mann–Whitney *U* test. LV: left ventricular.

**Table 3:** Primary and secondary outcomes in the matched cohort.

	Treatment Group (*n* = 59)	Control Group (*n* = 59)	*p*
Primary outcome			
ICU mortality	19 (32)	27 (46)	0.13
Secondary outcomes			
Vasopressor weaning	39 (66)	36 (61)	0.57
Vasopressor-free days at day 28	16.6 ± 12.1	15.0 ± 12.3	0.37
Ventilator weaning (*n* = 40/*n* = 35) ^1^	21 (53)	9 (26)	0.02
Ventilator-free days at day 28	11.4 ± 11.3	5.4 ± 9.7	0.02
Superinfection	6 (10)	13 (22)	0.08

Data are presented as number (%) or mean ± standard deviation. The *p*-values were calculated using the Mann–Whitney *U* test or Student’s *t*-test for continuous variables and using the chi-squared test or Fisher’s exact test for categorical variables. ICU: intensive care unit. ^1^ Mechanical ventilation was applied for 40 patients in the treatment group and for 35 patients in the control group.

**Table 4:** Association between the vitamin C protocol and ICU mortality.

	Crude		Propensity-Adjusted		PS-Matched		IPTW-Matched	
	Unadjusted HR (95% CI)	*p*	Adjusted HR (95% CI)	*p*	Adjusted HR (95% CI)	*p*	Adjusted HR (95% CI)	*p*
ICU mortality	0.57 (0.35–0.93)	0.02	0.60 (0.34–1.08)	0.09	0.55 (0.30–0.99)	0.047	0.67 (0.45–1.00)	0.048

The propensity-adjusted multivariable analysis was adjusted for lactate concentration and individual PS. The PS- and IPTW-matched multivariable analysis was adjusted for lactate concentration. CI: confidence interval; HR: hazard ratio; ICU: intensive care unit; IPTW: inverse probability of treatment weighting; PS: propensity score.

## Data Availability

Restrictions apply to the availability of these data. Data were obtained from electronic medical records in the Chung-Ang University Hospital database.

## Supplementary Materials

The following are available online at https://www.mdpi.com/article/10.3390/jpm11070610/s1, Table S1: Factors used to generate the propensity scores for the conditional probability that the patients would receive the vitamin C protocol; Table S2: Baseline characteristics of the groups before matching; Table S3: Primary and secondary outcomes in the groups before matching; Figure S1: Standardized mean differences for variables before and after matching and IPTW-trimming; Table S4: Echocardiographic parameters among patients with chronic heart failure or chronic lung disease; Table S5: Primary and secondary outcomes in the matched cohort according to the presence of chronic heart failure; Table S6: Primary and secondary outcomes in the matched cohort according to recovery status from septic cardiomyopathy; Table S7: Comparing primary and secondary outcomes among treated and control patients who did or did not receive steroids at shock onset; Table S8: Serial analyses of clinical parameters during the first 4 days in the matched cohort.
