# Supplementary material for: Early Vitamin C, Hydrocortisone, and Thiamine Treatment for Septic Cardiomyopathy: A Propensity Score Analysis
Source: J Pers Med. 2021 Jun 28;11(7):610. doi: 10.3390/jpm11070610 (PMC8304272; doi:10.3390/jpm11070610)
Supplement: Supplementary file 1 [file jpm-11-00610-s001.zip › jpm-1248138-supplementary.pdf]

Table S1. Factors used to generate the propensity scores for the conditional probability that the patients would receive the vitamin C protocol.

	**Odds ratio (95% confidence interval)**	***p***
Age	1.02 (0.99–1.06)	0.12
Male sex	2.91 (1.32–6.40)	0.01
Body mass index	0.99 (0.89–1.09)	0.78
Diabetes	0.56 (0.26–1.23)	0.15
Nosocomial infection	1.04 (0.45–2.37)	0.93
Pneumonia	0.50 (0.22–1.14)	0.10
SOFA score	0.93 (0.74–1.16)	0.50
Mechanical ventilation	3.22 (0.94–11.10)	0.06
Renal replacement therapy	1.53 (0.59–3.96)	0.38
Body temperature	0.69 (0.43–1.11)	0.13
Mean arterial pressure	1.00 (0.97–1.04)	0.86
Glasgow Coma Scale	0.99 (0.84–1.15)	0.85
$PaCO_2$	0.98 (0.94–1.03)	0.48
Bicarbonate	1.14 (1.03–1.26)	0.01
Potassium	0.77 (0.50–1.19)	0.24
Creatinine	0.90 (0.70–1.16)	0.42
Platelet count	1.00 (1.00–1.01)	0.71

The propensity scores were generated using variables with *p*-values of <0.20 (comparing treatment and control groups) and potential confounders that were judged based on clinical expertise. $PaCO_2$: arterial partial pressure of carbon dioxide; SOFA: Sequential Organ Failure Assessment.

Table S2. Baseline characteristics of the groups before matching.

	Treatment group (n = 91)	Control group (n = 75)	*p*
Age, years	77 (70–84)	73 (64–81)	0.054
Male sex	56 (62)	32 (43)	0.02
Body mass index, kg/m^2	20.0 (18.4–23.0)	21.2 (18.9–24.2)	0.04
Comorbidities			
Diabetes	29 (32)	35 (47)	0.051
Chronic heart failure	8 (9)	8 (11)	0.68
Chronic lung disease	15 (17)	10 (13)	0.57
Liver cirrhosis	7 (8)	8 (11)	0.51
Chronic kidney disease	21 (23)	20 (27)	0.59
Malignancy	18 (20)	10 (13)	0.27
Immunosuppression	16 (18)	17 (23)	0.41
Cause of sepsis			
Pneumonia	48 (53)	36 (48)	0.54
Urosepsis	22 (24)	23 (31)	0.35
Gastrointestinal/biliary	21 (23)	21 (28)	0.47
Nosocomial infection	29 (32)	24 (32)	0.99
ARDS	8 (9)	7 (9)	0.90
SOFA score	11 (10–14)	12 (9–14)	0.31
Mechanical ventilation	65 (71)	43 (57)	0.06
Renal replacement therapy	38 (42)	29 (39)	0.69
Vital signs & laboratory data			
Body temperature, °C	37.0 (36.7–37.9)	37.4 (36.9–38.0)	0.06
Mean arterial pressure, mmHg	61 (57–66)	59 (52–66)	0.18
Respiratory rate, breaths/min	28 (25–32)	28 (24–34)	0.81
Bicarbonate, mEq/L	18.7 (15.7–22.8)	16.1 (12.7–18.6)	<0.001
Creatinine, mg/dL	1.5 (1.0–2.2)	1.8 (1.2–2.6)	0.04
Platelet count, 1000/mm^3	140 (91–190)	112 (58–177)	0.07
Total bilirubin, mg/dL	0.8 (0.5–1.7)	0.7 (0.4–1.2)	0.27
C-reactive protein, mg/L	133 (76–230)	144 (89–250)	0.53
Lactate, mmol/L	3.9 (1.9–6.6)	3.6 (2.0–7.2)	0.61
Cardiac troponin I, ng/L	163 (63–689)	211 (92–752)	0.18
Brain natriuretic peptide, pg/mL	518 (247–955)	381 (153–1055)	0.45
Norepinephrine equivalent dose, µg/min	10.5 (5.3–21.2)	10.5 (5.4–21.2)	0.70
Echocardiography (n = 44 / n = 34) [1]			
Ejection fraction, %	37 (30–44)	39 (32–45)	0.59

Data are presented as number (%) or median (interquartile range). The *p*-values were calculated using the Mann-Whitney *U* test or Student's *t*-test for continuous variables and using the chi-squared test or Fisher's exact test for categorical variables. ARDS: acute respiratory distress syndrome; SOFA: Sequential Organ Failure Assessment. [1] Echocardiography was performed for 44 patients in the treatment group and for 34 patients in the control group.

Table S3. Primary and secondary outcomes in the groups before matching.

	**Treatment group (n = 91)**	**Control group (n = 75)**	*p*
Primary outcome			
ICU mortality	33 (36)	34 (45)	0.24
Secondary outcomes			
Vasopressor weaning	60 (66)	45 (61)	0.50
Vasopressor-free days at day 28	16.5 ± 12.1	14.7 ± 12.3	0.26
Ventilator weaning (n = 65 / n = 43) [1]	30 (46)	11 (26)	0.03
Ventilator-free days at day 28	9.0 ± 10.5	5.4 ± 9.5	0.06
Superinfection	13 (14)	15 (20)	0.33

Data are presented as number (%) or mean ± standard deviation. The *p*-values were calculated using the Mann-Whitney *U* test or Student's *t*-test for continuous variables and using the chi-squared test or Fisher's exact test for categorical variables. ICU: intensive care unit. [1] Mechanical ventilation was applied for 65 patients in the treatment group and for 43 patients in the control group.

Figure S1. Standardized mean differences for variables before and after matching and IPTW-trimming.

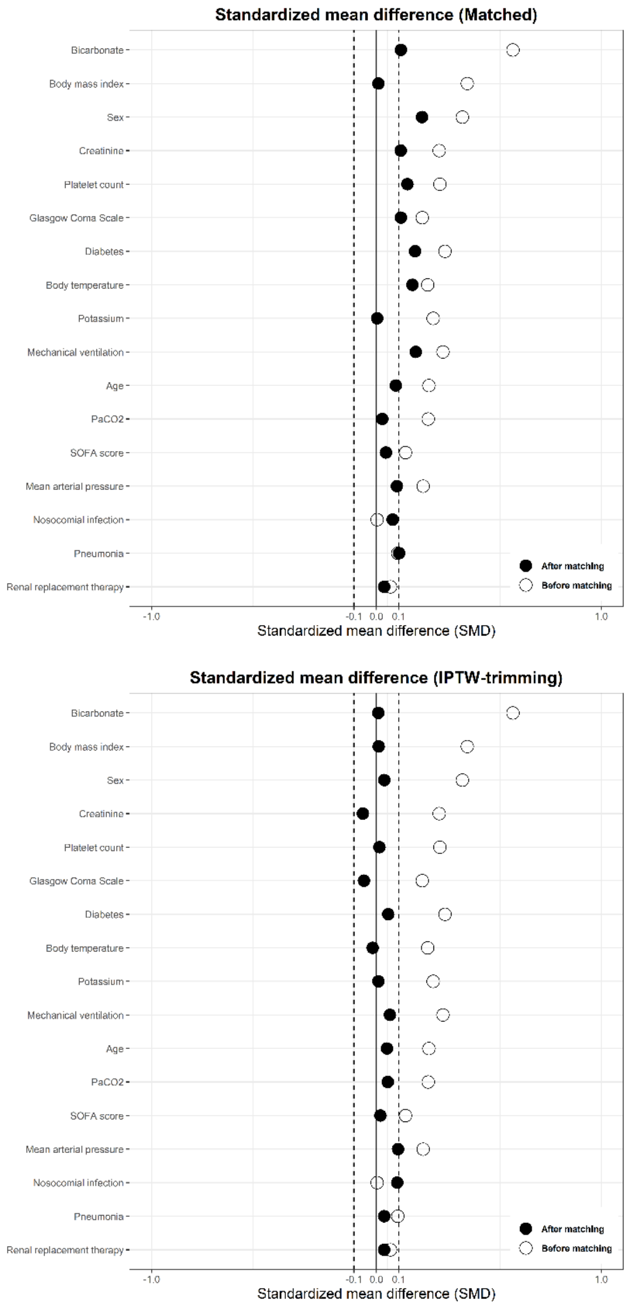

IPTW: inverse probability of treatment weighting; $PaCO_2$: arterial partial pressure of carbon dioxide; SOFA: Sequential Organ Failure Assessment.

Table S4. Echocardiographic parameters among patients with chronic heart failure or chronic lung disease.

	**Treatment group**	**Control group**	***p***
Chronic heart failure	n = 4	n = 3	
LV ejection fraction, %	29 (24–36)	41 (34–43)	0.16
Mitral *E* wave, cm/s	71.0 (52.5–85.0)	105.0 (78.0–122.5)	0.29
Mitral *E′* wave, cm/s	4.0 (4.0–4.5)	5.0 (4.0–8.0)	0.58
E/*E′* ratio	16.2 (13.9–20.1)	14.0 (13.4–17.8)	0.72
Stroke volume, mL	36 (29–38)	30 (29–37)	>0.99
Cardiac output, L/min	2.8 (2.3–3.1)	3.7 (2.9–3.8)	0.29
Cardiac index, L/min/m^2	1.8 (1.4–2.1)	2.4 (2.0–2.5)	0.29
Chronic lung disease	n = 3	n = 5	
LV ejection fraction, %	24 (22–28)	39 (37–39)	0.10
Mitral *E* wave, cm/s	95.0 (69.0–99.0)	73.0 (65.0–95.0)	>0.99
Mitral *E′* wave, cm/s	6.0 (5.0–6.5)	5.0 (5.0–6.0)	0.76
E/*E′* ratio	13.6 (12.7–16.1)	13.0 (12.6–21.5)	0.88
Stroke volume, mL	35 (27–36)	27 (24–28)	0.88
Cardiac output, L/min	2.8 (2.7–3.0)	3.3 (3.2–3.4)	0.07
Cardiac index, L/min/m^2	1.7 (1.7–1.9)	2.2 (2.1–2.6)	0.04

Data are presented as median (interquartile range). The *p*-values were calculated using the Mann-Whitney *U* test. LV: left ventricular.

Table S5. Primary and secondary outcomes in the matched cohort according to the presence of chronic heart failure.

	**Chronic heart failure (n = 12)**	**No chronic heart failure (n = 106)**	***p***
Primary outcome			
ICU mortality	5 (42)	40 (38)	0.77
Secondary outcomes			
Vasopressor weaning	7 (58)	68 (64)	0.76
Vasopressor-free days at day 28	14.8 ± 13.1	15.8 ± 12.1	0.32
Ventilator weaning (n = 9 / n = 64) [1]	4 (44)	26 (41)	>0.99
Ventilator-free days at day 28	10.6 ± 12.6	8.5 ± 10.6	0.02
Superinfection	1 (8)	16 (15)	>0.99

Data are presented as number (%) or mean ± standard deviation. The *p*-values were calculated using the Mann-Whitney *U* test or Student's *t*-test for continuous variables and using the chi-squared test or Fisher's exact test for categorical variables. ICU: intensive care unit. [1] Mechanical ventilation was applied for 9 patients in the chronic heart failure group and for 64 patients in the no chronic heart failure group.

Table S6. Primary and secondary outcomes in the matched cohort according to recovery status from septic cardiomyopathy.

	Recovery (n = 17)	No recovery (n = 7)	*p*
Primary outcome			
ICU mortality	1 (6)	3 (43)	0.059
Secondary outcomes			
Vasopressor weaning	16 (94)	3 (43)	0.01
Vasopressor-free days at day 28	23.5 ± 6.3	10.7 ± 13.5	0.08
Ventilator weaning (n = 11 / n = 5) [1]	9 (82)	2 (40)	0.25
Ventilator-free days at day 28	16.3 ± 8.9	10.0 ± 13.7	0.73
Superinfection	3 (18)	1 (14)	>0.99

Data are presented as number (%) or mean ± standard deviation. The *p*-values were calculated using the Mann-Whitney *U* test or Student's *t*-test for continuous variables and using the chi-squared test or Fisher's exact test for categorical variables. ICU: intensive care unit. [1] Mechanical ventilation was applied for 11 patients in the recovery group and for 5 patients in the no recovery group.

Table S7. Comparing primary and secondary outcomes among treated and control patients who did and did not receive steroids at shock onset.

	**Treatment group (n = 59)**	**Control group with steroids (n = 13)**	**Control group without steroids (n = 46)**	***p***
Primary outcome				
ICU mortality	19 (32)	8 (62)	18 (39)	0.14
Secondary outcomes				
Vasopressor weaning	39 (66)	6 (46)	30 (65)	0.38
Vasopressor-free days at day 28	16.6 ± 12.1	11.5 ± 13.0	15.9 ± 12.0	0.34
Ventilator weaning (n = 40 / n = 10 / n = 23) [1]	21 (53)	2 (20)	7 (30)	0.08
Ventilator-free days at day 28	11.5 ± 11.3	3.8 ± 8.1	6.0 ± 9.7	0.045
Superinfection	6 (10)	2 (15)	9 (20)	0.39

Data are presented as number (%) or mean ± standard deviation. The *p*-values were calculated using the Kruskal-Wallis test for continuous variables and using the chi-squared test or Fisher's exact test for categorical variables. ICU: intensive care unit. [1] Mechanical ventilation was applied for 40 patients in the treatment group, 10 patients in the control group with steroids, and 23 patients in the control group without steroids.

Table S8. Serial analyses of clinical parameters during the first 4 days in the matched cohort.

	**Treatment group (n = 59)**	**Control group (n = 59)**	***p***
Day 2 (n = 59 / n = 53)			
Mean arterial pressure, mmHg	62 (52–66)	60 (56–66)	0.85
Creatinine, mg/dL	1.3 (1.0–2.1)	1.3 (0.8–2.1)	0.62
Platelet count, 1000/mm^3	103 (68–163)	108 (61–182)	0.97
Total bilirubin, mg/dL	0.8 (0.5–2.2)	0.6 (0.4–1.2)	0.04
C-reactive protein, mg/L	226 (137–280)	189 (124–288)	0.60
Lactate, mmol/L	3.8 (2.1–6.6)	2.7 (1.9–6.2)	0.19
Glucose, mg/dL	182 (135–238)	174 (147–222)	0.93
Norepinephrine equivalent dose, μg/min	7.7 (0–19.1)	9.7 (3.0–30.7)	0.29
SOFA score	12 (9–14)	12 (8–14)	0.88
Net fluid retention [1], mL	795 (375–1410)	1291 (318–2030)	0.12
Day 3 (n = 54 / n = 44)			
Mean arterial pressure, mmHg	64 (52–72)	59 (54–67)	0.40
Creatinine, mg/dL	1.2 (0.8–1.7)	1.0 (0.7–1.7)	0.46
Platelet count, 1000/mm^3	85 (43–149)	88 (41–159)	0.93
Total bilirubin, mg/dL	1.0 (0.5–1.9)	0.6 (0.4–1.2)	0.04
C-reactive protein, mg/L	173 (73–253)	211 (126–322)	0.04
Lactate, mmol/L	2.7 (2.0–4.8)	2.6 (1.6–7.3)	0.35
Glucose, mg/dL	173 (139–212)	179 (135–213)	0.89
Norepinephrine equivalent dose, μg/min	1.9 (0–12.8)	8.4 (0–18.8)	0.07
SOFA score	10 (7–14)	12 (8–18)	0.37
Net fluid retention [1], mL	530 (–86 to 1010)	1158 (610–2374)	<0.001
Day 4 (n = 46 / n = 36)			
Mean arterial pressure, mmHg	67 (55–81)	60 (56–69)	0.26
Creatinine, mg/dL	1.1 (0.6–1.5)	1.0 (0.6–1.6)	0.82
Platelet count, 1000/mm^3	77 (50–145)	77 (41–154)	0.89
Total bilirubin, mg/dL	1.0 (0.6–1.8)	0.6 (0.4–1.4)	0.08
C-reactive protein, mg/L	106 (60–208)	199 (124–271)	<0.001
Lactate, mmol/L	3.0 (1.8–4.8)	2.4 (1.3–5.9)	0.19
Glucose, mg/dL	162 (128–192)	155 (123–212)	0.91
Norepinephrine equivalent dose, μg/min	0 (0–10.5)	4.4 (0–21.2)	0.08
SOFA score	10 (6–15)	11 (6–24)	0.40
Net fluid retention [1], mL	221 (–235 to 1053)	765 (–113 to 1646)	0.08
Change relative to day 1			
C-reactive protein, day 4	–25 (–116 to 46)	0 (–30 to 122)	0.004
Norepinephrine equivalent dose, day 4	–8.4 (–21.0 to 0)	–4.1 (–10.8 to 2.0)	0.04
SOFA score, day 4	–2 (–5 to 1)	–1 (–3 to 4)	0.09

Data are presented as median (interquartile range). The *p*-values were calculated using the Mann-Whitney *U* test or Student's *t*-test. SOFA: Sequential Organ Failure Assessment. [1] Net fluid retention was calculated as the difference between all fluid intake and all fluid output (urine volume, dialysis volume, drainage volume, and stool weight).
